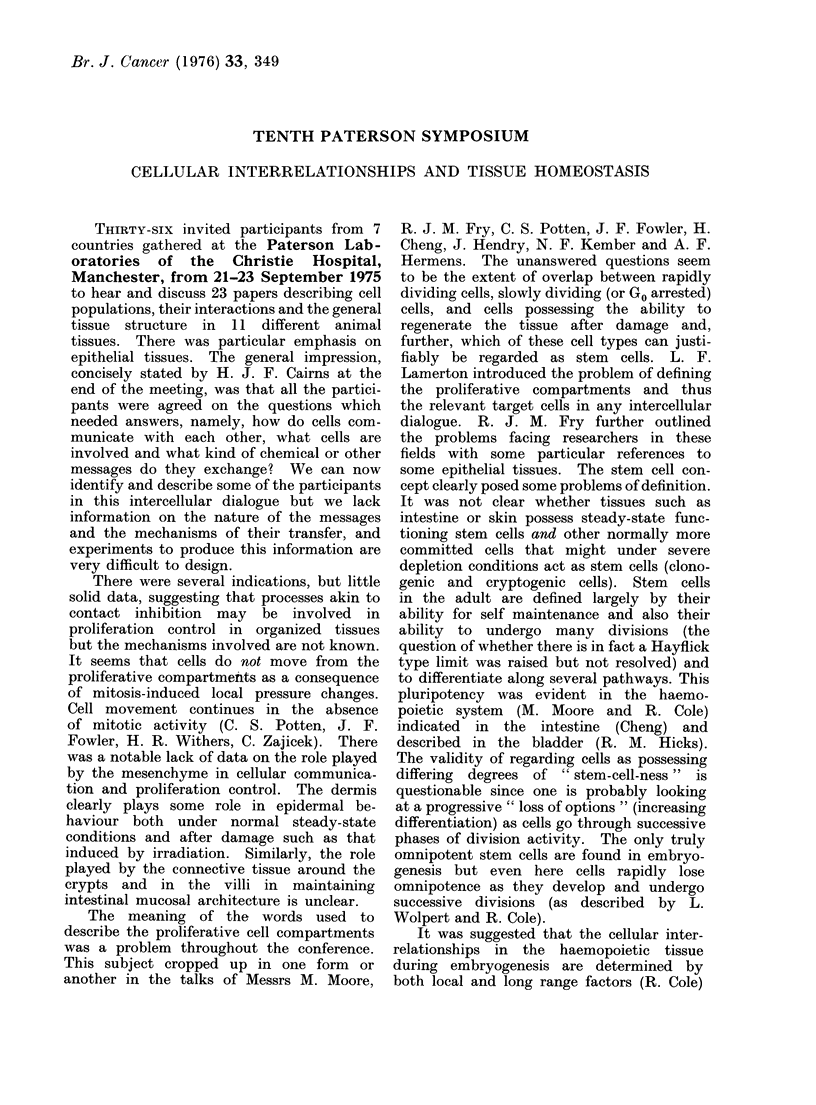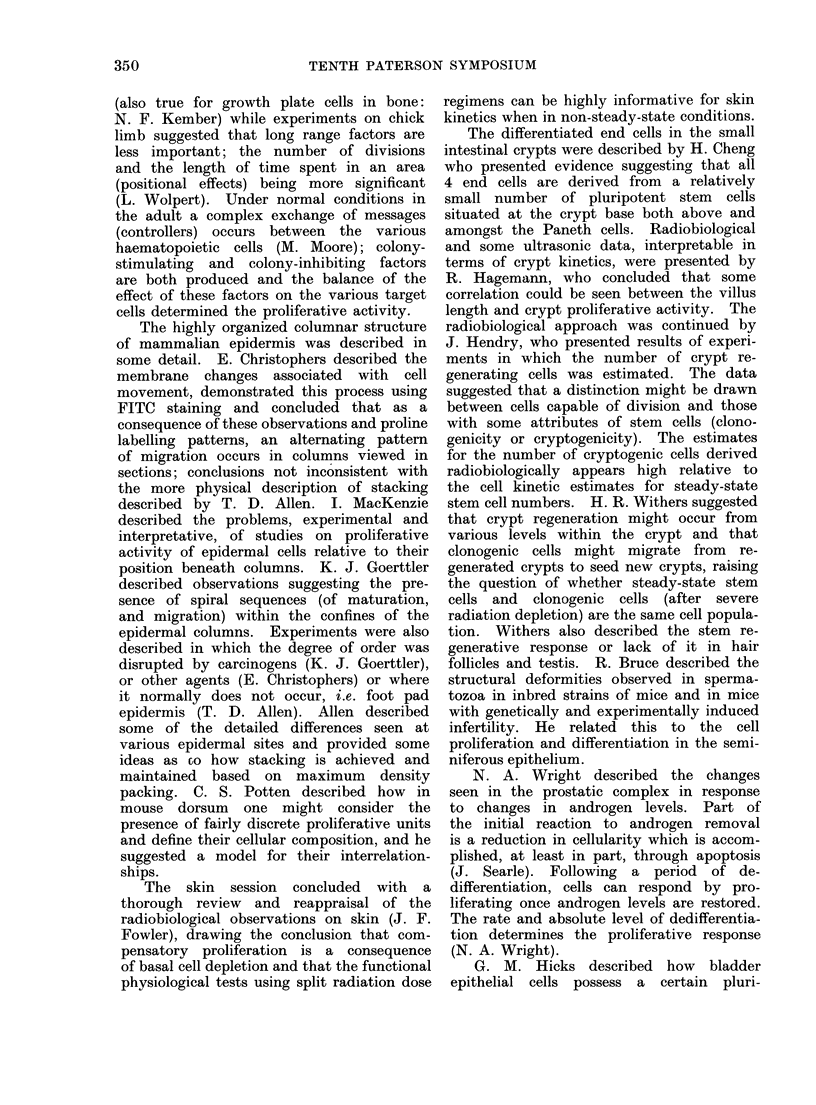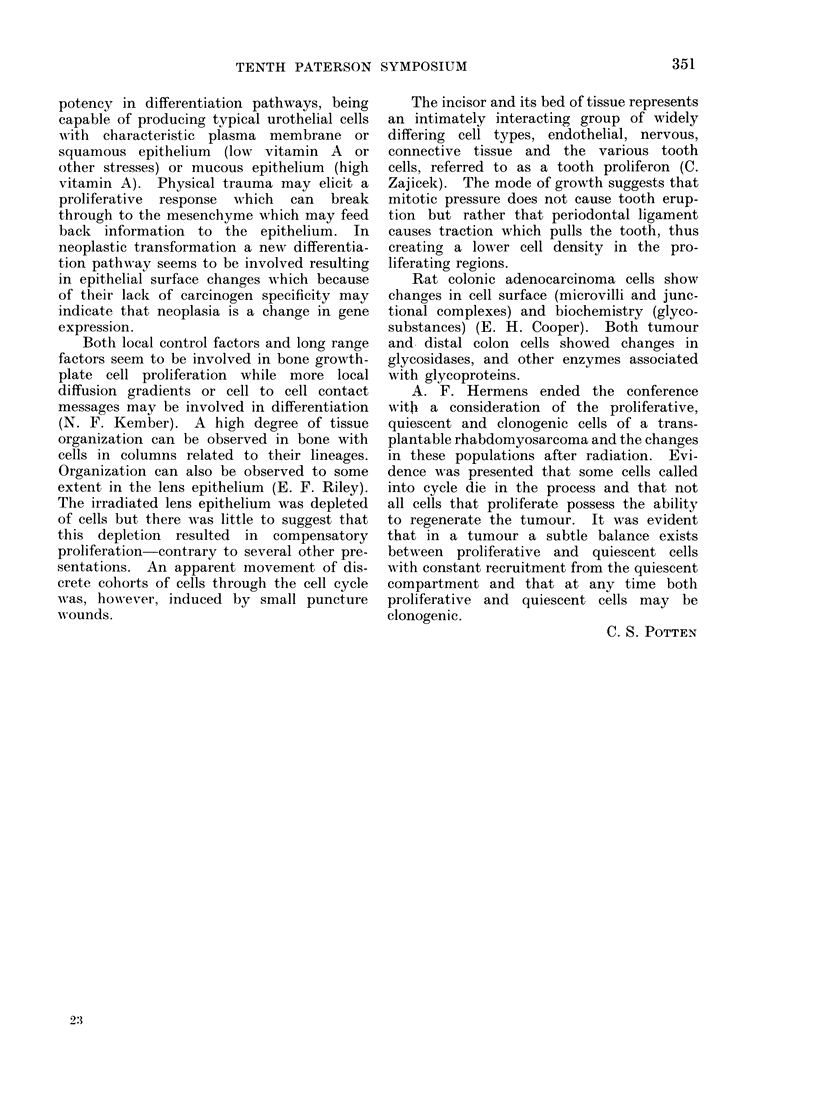# Tenth Paterson Symposium

**Published:** 1976-03

**Authors:** C. S. Potten


Br. J. Cancer (1976) 33, 349

### TENTH PATERSON SYMPOSIUM

### CELLULAR INTERRELATIONSHIPS AND TISSUE HOMEOSTASIS

THIRTY-SIX invited participants from 7 countries gathered at the **Paterson Laboratories of the Christie Hospital, Manchester, from 21–23 September 1975** to hear and discuss 23 papers describing cell populations, their interactions and the general tissue structure in 11 different animal tissues. There was particular emphasis on epithelial tissues. The general impression, concisely stated by H. J. F. Cairns at the end of the meeting, was that all the participants were agreed on the questions which needed answers, namely, how do cells communicate with each other, what cells are involved and what kind of chemical or other messages do they exchange? We can now identify and describe some of the participants in this intercellular dialogue but we lack information on the nature of the messages and the mechanisms of their transfer, and experiments to produce this information are very difficult to design.

There were several indications, but little solid data, suggesting that processes akin to contact inhibition may be involved in proliferation control in organized tissues but the mechanisms involved are not known. It seems that cells do *not* move from the proliferative compartments as a consequence of mitosis-induced local pressure changes. Cell movement continues in the absence of mitotic activity (C. S. Potten, J. F. Fowler, H. R. Withers, C. Zajicek). There was a notable lack of data on the role played by the mesenchyme in cellular communication and proliferation control. The dermis clearly plays some role in epidermal behaviour both under normal steady-state conditions and after damage such as that induced by irradiation. Similarly, the role played by the connective tissue around the crypts and in the villi in maintaining intestinal mucosal architecture is unclear.

The meaning of the words used to describe the proliferative cell compartments was a problem throughout the conference. This subject cropped up in one form or another in the talks of Messrs M. Moore, R. J. M. Fry, C. S. Potten, J. F. Fowler, H. Cheng, J. Hendry, N. F. Kember and A. F. Hermens. The unanswered questions seem to be the extent of overlap between rapidly dividing cells, slowly dividing (or $G_0$ arrested) cells, and cells possessing the ability to regenerate the tissue after damage and, further, which of these cell types can justifiably be regarded as stem cells. L. F. Lamerton introduced the problem of defining the proliferative compartments and thus the relevant target cells in any intercellular dialogue. R. J. M. Fry further outlined the problems facing researchers in these fields with some particular references to some epithelial tissues. The stem cell concept clearly posed some problems of definition. It was not clear whether tissues such as intestine or skin possess steady-state functioning stem cells *and* other normally more committed cells that might under severe depletion conditions act as stem cells (clonogenic and cryptogenic cells). Stem cells in the adult are defined largely by their ability for self maintenance and also their ability to undergo many divisions (the question of whether there is in fact a Hayflick type limit was raised but not resolved) and to differentiate along several pathways. This pluripotency was evident in the haemopoietic system (M. Moore and R. Cole) indicated in the intestine (Cheng) and described in the bladder (R. M. Hicks). The validity of regarding cells as possessing differing degrees of "stem-cell-ness" is questionable since one is probably looking at a progressive "loss of options" (increasing differentiation) as cells go through successive phases of division activity. The only truly omnipotent stem cells are found in embryogenesis but even here cells rapidly lose omnipotence as they develop and undergo successive divisions (as described by L. Wolpert and R. Cole).

It was suggested that the cellular interrelationships in the haemopoietic tissue during embryogenesis are determined by both local and long range factors (R. Cole)

(also true for growth plate cells in bone: N. F. Kember) while experiments on chick limb suggested that long range factors are less important; the number of divisions and the length of time spent in an area (positional effects) being more significant (L. Wolpert). Under normal conditions in the adult a complex exchange of messages (controllers) occurs between the various haematopoietic cells (M. Moore); colony-stimulating and colony-inhibiting factors are both produced and the balance of the effect of these factors on the various target cells determined the proliferative activity.

The highly organized columnar structure of mammalian epidermis was described in some detail. E. Christophers described the membrane changes associated with cell movement, demonstrated this process using FITC staining and concluded that as a consequence of these observations and proline labelling patterns, an alternating pattern of migration occurs in columns viewed in sections; conclusions not inconsistent with the more physical description of stacking described by T. D. Allen. I. MacKenzie described the problems, experimental and interpretative, of studies on proliferative activity of epidermal cells relative to their position beneath columns. K. J. Goerttler described observations suggesting the presence of spiral sequences (of maturation, and migration) within the confines of the epidermal columns. Experiments were also described in which the degree of order was disrupted by carcinogens (K. J. Goerttler), or other agents (E. Christophers) or where it normally does not occur, *i.e.* foot pad epidermis (T. D. Allen). Allen described some of the detailed differences seen at various epidermal sites and provided some ideas as co how stacking is achieved and maintained based on maximum density packing. C. S. Potten described how in mouse dorsum one might consider the presence of fairly discrete proliferative units and define their cellular composition, and he suggested a model for their interrelationships.

The skin session concluded with a thorough review and reappraisal of the radiobiological observations on skin (J. F. Fowler), drawing the conclusion that compensatory proliferation is a consequence of basal cell depletion and that the functional physiological tests using split radiation dose regimens can be highly informative for skin kinetics when in non-steady-state conditions.

The differentiated end cells in the small intestinal crypts were described by H. Cheng who presented evidence suggesting that all 4 end cells are derived from a relatively small number of pluripotent stem cells situated at the crypt base both above and amongst the Paneth cells. Radiobiological and some ultrasonic data, interpretable in terms of crypt kinetics, were presented by R. Hagemann, who concluded that some correlation could be seen between the villus length and crypt proliferative activity. The radiobiological approach was continued by J. Hendry, who presented results of experiments in which the number of crypt regenerating cells was estimated. The data suggested that a distinction might be drawn between cells capable of division and those with some attributes of stem cells (clonogenicity or cryptogenicity). The estimates for the number of cryptogenic cells derived radiobiologically appears high relative to the cell kinetic estimates for steady-state stem cell numbers. H. R. Withers suggested that crypt regeneration might occur from various levels within the crypt and that clonogenic cells might migrate from regenerated crypts to seed new crypts, raising the question of whether steady-state stem cells and clonogenic cells (after severe radiation depletion) are the same cell population. Withers also described the stem regenerative response or lack of it in hair follicles and testis. R. Bruce described the structural deformities observed in spermatozoa in inbred strains of mice and in mice with genetically and experimentally induced infertility. He related this to the cell proliferation and differentiation in the seminiferous epithelium.

N. A. Wright described the changes seen in the prostatic complex in response to changes in androgen levels. Part of the initial reaction to androgen removal is a reduction in cellularity which is accomplished, at least in part, through apoptosis (J. Searle). Following a period of dedifferentiation, cells can respond by proliferating once androgen levels are restored. The rate and absolute level of dedifferentiation determines the proliferative response (N. A. Wright).

G. M. Hicks described how bladder epithelial cells possess a certain pluripotency in differentiation pathways, being capable of producing typical urothelial cells with characteristic plasma membrane or squamous epithelium (low vitamin A or other stresses) or mucous epithelium (high vitamin A). Physical trauma may elicit a proliferative response which can break through to the mesenchyme which may feed back information to the epithelium. In neoplastic transformation a new differentiation pathway seems to be involved resulting in epithelial surface changes which because of their lack of carcinogen specificity may indicate that neoplasia is a change in gene expression.

Both local control factors and long range factors seem to be involved in bone growth-plate cell proliferation while more local diffusion gradients or cell to cell contact messages may be involved in differentiation (N. F. Kember). A high degree of tissue organization can be observed in bone with cells in columns related to their lineages. Organization can also be observed to some extent in the lens epithelium (E. F. Riley). The irradiated lens epithelium was depleted of cells but there was little to suggest that this depletion resulted in compensatory proliferation—contrary to several other presentations. An apparent movement of discrete cohorts of cells through the cell cycle was, however, induced by small puncture wounds.

The incisor and its bed of tissue represents an intimately interacting group of widely differing cell types, endothelial, nervous, connective tissue and the various tooth cells, referred to as a tooth proliferon (C. Zajicek). The mode of growth suggests that mitotic pressure does not cause tooth eruption but rather that periodontal ligament causes traction which pulls the tooth, thus creating a lower cell density in the proliferating regions.

Rat colonic adenocarcinoma cells show changes in cell surface (microvilli and junctional complexes) and biochemistry (glycosubstances) (E. H. Cooper). Both tumour and distal colon cells showed changes in glycosidases, and other enzymes associated with glycoproteins.

A. F. Hermens ended the conference with a consideration of the proliferative, quiescent and clonogenic cells of a transplantable rhabdomyosarcoma and the changes in these populations after radiation. Evidence was presented that some cells called into cycle die in the process and that not all cells that proliferate possess the ability to regenerate the tumour. It was evident that in a tumour a subtle balance exists between proliferative and quiescent cells with constant recruitment from the quiescent compartment and that at any time both proliferative and quiescent cells may be clonogenic.

C. S. POTTEN